# The Treatment of Suppuration

**Published:** 1919-04-26

**Authors:** E. G. Slesinger


### THE TREATMENT OF SUPPURATION.

### With a Note on Maggots.

By E. G. SLESINGER, M.B., B.S., B.Sc.(LOND.), F.R.C.S.(ENG.),

During the present war wounds caused by fragments of shell or bomb have been considerably more frequent than wounds caused by the high velocity, and therefore relatively sterile, bullet, and consequently the majority of all severe wounds have proved to be heavily infected from the moment of their infliction. As a result an enormous number of suppurating wounds have come under observation and have enabled valuable observations to be made as to their treatment. Such infected wounds have formed in many ways a unique field for observation, partly because the occurrence of large infected wounds, where direct observation is much easier than in the trunk, is rare in civil work; partly because the infection in these cases was usually a sudden infection implanted directly on to previously healthy tissues; and partly because the occurrence of cases in bulk enabled comparative results under equitable conditions to be readily obtained.

### Methods in Use.

The conditions in which suppuration occurs in civil life are, in most cases, not strictly comparable to those obtaining in war wounds, particularly in that the latter is usually an implantation infection on to normally re-acting tissues, while the former represents in most cases the result of a disturbance in the balance between attack and defence in the body, resulting, in Wright's terminology, in deficient kataphylacis. Therefore inferences drawn from war experiences are not directly transferable to the consideration of sepsis in civil life, but at the same time the principles underlying the treatment of the two groups remain the same, and the lessons of the war should provide a satisfactory basis for further advance. In the main the lines along which an attempt has been made to cope with suppuration in war wounds have been three in number—namely (1) The use of antiseptics, (2) the so-called "physiological" method, and (3) the removal of dead tissues, or what may be termed the "starvation" method.

### Antiseptics.

The use of antiseptics in a suppurating wound has on the whole been uniformly disappointing, partly no doubt from the fact that too much was expected from their action, but at the same time such a result is not surprising when the objects which it is desired to achieve are kept in view. The purpose for which antiseptics are employed in a wound is to kill the infecting microbes; and consequently an ideal antiseptic requires to satisfy several essential conditions, which are mainly (*a*) that it shall destroy organisms without destroying living tissue, (*b*) that it shall carry out its germicidal action in the presence of the substances normally present in the wound—*e.g.*, serum, etc.—and (*c*) that it shall be able to *reach* all the microbes in the wound in order that it may carry out its desired action. Now, even if the first two conditions prove capable of fulfilment—and considering the close relationship between microbes and cells as units, such a hope seems singularly optimistic—the need of reaching to all parts of the wound appears an insuperable difficulty. The wall of a wound during its infected stage is lined by a layer of dead tissue, and it is beneath this layer that the infecting microbes are essentially at work. Organisms in the cavity of the wound are already outside the body, and their death is of purely secondary importance, a fact of which the usual laboratory antiseptic tests take no notice at all. An attempt was made to overcome this difficulty in the manner in which B.I.P.P. was employed, and the explanation of its failure to secure more than partial success would seem to lie in its inability to fulfil the first two conditions.

### Physiological Method.

The second, or "physiological," method aims at reproducing the normal method of natural cure in suppuration—namely, by encouraging the emigration of leucocytes, of aiding their destruction—thereby increasing the liberation of trypsin in the wound and consequently of producing a digestive cleansing of the surface as does Nature herself. On these bases was built up the treatment of wounds by the salt method, and by it it was hoped to flush the wounds from within outwards by the body's own fluids, to increase the trypsin production in the wound, or in other words, to hasten and aid the mobilisation of the natural means of defence. That this method proved successful in a number of cases there can be no doubt, and it would seem that its failure in others was mainly due to the inability of the salt to carry out its desired action owing to the dead tissue interposed between it and the actual wall of the wound.

### Starvation Method.

The third method—the "starvation" method—aims at securing the same result, but instead of relying on the supply of digestive or hydrolytic substance from within the body, attempts to secure its introduction from without. In this way it is hoped to destroy the dead tissue lining of the wound, and consequently to remove the medium from the microbes, instead of, as in the antiseptic method, attempting to remove the microbes from the medium.

### Action of Maggots.

Many methods of securing this desirable result have been attempted, and in this connection an experiment which frequently occurs in the course of nature is worthy of note. In many patients who had been exposed for some time after the receipt of their injury, the wound proved to be infected with the maggot of the common fly, and indeed in such campaigns as that in Gallipoli such an infection was rather the rule than the exception. When such a case reached the surgeon it was found that although the wound was foully malodorous, on removal of the maggots the surface proved, to be comparatively clean and covered with healthy granulations. The larger and more numerous the maggots the cleaner the surface, and such a maggoty wound could frequently be subjected to secondary suture within a day or two after it was first seen. This method of removing dead tissue has undoubted æsthetic objections, but at the same time it often afforded the patient a method of successful treatment when more cleanly and less offensive ones were beyond his reach.

### Hydrolytic Agents.

The more usual methods of securing the removal of dead tissues from the wound consist in the employment of hydrolytic agents, which may be chemical, bacterial, or fermentative in nature. Of bacterial agents, the so-called Reading bacillus has been employed for the purpose by Robertson, and it is claimed for this organism that while non-pathogenic to man, it rapidly hydrolyses dead tissues.

Of the chemical hydrolytic agents the most successful has proved to be hypochlorite solution. This solution would appear to act solely by virtue of its action on protein, its laboratory antiseptic power being inactive in the wound on account of its immediate destruction when in contact with dissolved protein. It rapidly hydrolyses protein in solution down to amino acids, and by its use in the form of intermittent irrigation the wound is speedily cleaned of its dead tissue, and the invading organisms, deprived of their medium for growth, rapidly disappear. The use of ferments to achieve the same purpose has not as yet been extensively tried, partly because of expense, and partly because of the difficulty of guiding and limiting their action, but their further study would seem to offer prospects of considerable value.

### Defence Reaction.

In addition to the digestive cleansing of the wound surface the natural reactions of the body in response to infection consist of an attempt to prevent the spread of the invading microbe, partly by the construction of a zone of leucocytic infiltration round the wound and partly by the increase of the normal antityptic reaction of the serum. The importance of the intactness of the zone of leucocytic infiltration in the prevention of septicæmia cannot be overestimated, and there can be little doubt that the usual exciting cause of septicæmia in war cases was the violation of this zone either by insufficient outlets for pus, or by the muscular movements consequent on inefficient splinting, or by too extensive surgical procedures in search of hidden pus. The alterations in the antityptic reaction in response to infection would appear to be of far greater importance than is at present thought, and may possibly offer the reason for the differences in the results of infections which the variations of virulence in the organism are not sufficient to explain alone, a question which is the subject of further investigation at present. While, as has been already pointed out, many differences are operative between suppuration in military and civilian surgery, yet the application of the lessons learnt from the former holds out undoubted promise of improvement in the treatment of the latter; and the secondary suture which so frequently followed the successful application of the starvation method of wound treatment should prove of inestimable value in obviating the disabilities which so often follow suppuration in civil cases. As to what method of securing the desired result will prove to be most applicable remains to be seen, but the experiments of war have at any rate been of value in that they have shown that by the hydrolytic removal of dead tissue it is possible to secure the rapid cleansing of a suppurating wound, which can then be safely and satisfactorily closed as a secondary operation.

